# Immune priming with inactive dengue virus during the larval stage of *Aedes aegypti* protects against the infection in adult mosquitoes

**DOI:** 10.1038/s41598-020-63402-z

**Published:** 2020-04-21

**Authors:** Valeria Vargas, Jorge Cime-Castillo, Humberto Lanz-Mendoza

**Affiliations:** 1 National Institute of Public Health, Center for Research on Infectious Diseases, Santa María Ahuacatitlán, Cuernavaca, 62100 Morelos Mexico; 2 Postgraduate in Biological Sciences, National Autonomous University of Mexico, Av. Ciudad Universitaria 3000, Coyoacán, C.P. 04510 Mexico

**Keywords:** Viral infection, RNAi

## Abstract

Several studies have observed that the immune response in insects can be conserved, a phenomenon known as immune priming, which has been mostly tested in adult stages. However, it is unknown if induction of immune priming in larval stages protects against dengue virus (DENV) infections in adult mosquitoes. In this work, we primed larval instar 3^rd^ of *Aedes aegypti* with inactive dengue virus, producing adult mosquitoes with i) an enhanced antiviral-immune response; ii) a reduction in the load and replication of RNA of dengue virus (DENV); iii) a decline in viral infective particles production. Adult mosquitoes previously primed during larval stages over-expressed RNA interference (RNAi) markers Argonaute-2 (*AGO-2*) and Dicer-2 (*DCR-2*). We also observed inter-individual variations of DENV infection in adult mosquitoes, indicating a heterogeneous response to DENV infection in the same mosquito strain. However, mosquitoes primed during larval stages appear to control the infection, reducing the viral load. The over-expression of interferon-like factors (*VAGO*) and *AGO-2* in the pupa stage suggests a fast activation of antiviral mechanisms after immune priming in larvae, creating a condition in which adult mosquitoes are resistant to the pathogen in the posterior exposure.

## Introduction

*Aedes aegypti* is the primary vector of dengue virus (DENV). It is responsible for an estimated 390 million human infections each year^1,2^. The vaccine lacking to prevent the infection in humans, and the mosquito resistance to different insecticides, make it urgent to develop new strategies to interrupt the transmission by this mosquito^3,4^. Immune priming has become an important strategy to induce a protective condition against different pathogens in insects, occurring when a primary infectious exposure leads to an immune response that is enhanced upon re-exposure. This phenomenon has been described in various invertebrate taxa, including mosquitoes^3,4^. Recent work by our group has analyzed the ability of *Ae. aegypti* to mount enhanced antiviral responses upon secondary oral DENV immune challenges. Following priming with a live or inactive virus, secondary DENV infections were shown to diminish in midguts and carcasses^5^. Also, higher *de novo* midgut DNA synthesis was detected in challenged tissues, as determined by the incorporation of 5-bromo-2-deoxyuridine (BrDU) in DNA. Endoreplication, a type of *de novo* DNA synthesis, was shown to correlate with the activation of the Notch pathway, where *Hindsight*, *Delta*, and *Notch* were overexpressed after a secondary challenge with active DENV, in primed *Ae. aegypti* mosquitoes^6^. These findings open a new perspective on the mechanisms underlying the vector’s antiviral immune response and the effector molecules involved. Similar results have been observed in *Anopheles albimanus*^7^, in the LSB-AA695BB cell line (*An. albimanus*) and trained monocytes^8^. The induction of this immune response could be difficult to carry out in the field. Therefore, it is essential to determine if we can activate the immune response in other developmental stages (ontogeny stages).

The larval stage is an attractive target because of its aquatic requirements. Recently, we observed that the induction of immune priming against *Escherichia coli* could be generated in the larval stage in *Ae. aegypti*^9^.

Susceptibility of *Ae. aegypti* and *Ae. albopictus* larvae to Dengue virus infection has been demonstrated for three different serotypes^10^. These results indicate that immune priming at the larval stage with inactive DENV (*inact-DV*), could enhance the antiviral immune response in adult mosquitoes against a second challenge with active DENV (*act-DV*). Currently, few studies have evaluated the induction of immune priming in early development stages in insects^11–16^. On the other hand, it has been suggested that *Ae. aegypti* is tolerant of dengue infection and this tolerance allows the dengue virus to overcome the mosquito immune response; therefore allowing the transmission^17,18^. In the present study, we induced a protective condition in adult mosquitoes after priming 3^rd^ instar larvae with *inact-DV*, serotype 4. The dynamic analysis of the infection after the second challenge in primed (*Pr*) and unprimed (*UnPr*) individual adult mosquitoes was evaluated using individual excreta as recently described by Fontaine *et al*.,^19^. We evaluated the antiviral immune response by analyzing the relative expression of small- interfering RNAs (*siRNAs*) markers, like Argonaute-2 (*AGO-2*), Dicer-2 (*DCR-2*) and *R2D2*^20,21^, as well as interferon-like factor *VAGO*^22,23^. We evaluated the siRNAs pathway without ruling out that other immune pathways might be involved.

## Results

### Establishment of the optimal virus concentration for the immune priming

The experimental design consisted of two steps (see Supplementary Fig. S1): immune priming (1^st^ challenge) was done in 3^rd^ instar larvae, and the second challenge (2^nd^ challenge) was done in adult female mosquitoes (3–5 days post-emergence). Larvae were incubated with *inact-DV* (2.1 × 10^5^ PFU/ml inactivated by UV-light for 1 hour at 4 °C) in 198 ml of sterile tap water for 24 h. In order to establish the best dengue virus concentration for immune priming, 3^th^ instar larvae (*N* = 10 larvae per treatment) were exposed to a serial dilution of *act-DV* (10^6^–10^1^; see Supplementary Fig. S2) and the viral effect was evaluated through the relative expression of immune gene markers for Defensin, Cecropin, Attacin, Cactus and Relish 1. Larvae exposed to *act-DV* (2.1 × 10^5^ PFU/ml) for 24 h activated the immune response markers in *Ae. aegypti* (see Supplementary Fig. S2). Also, we evaluated different vehicle mediums, observing that sterile tap water was the best vehicle for infection in the larval stage (MEM, PBS 1×, 0.1% saline solution and sterilized tap water; see Supplementary Fig. S3).

### Immune priming in *Ae. aegypti* third instar larvae with *inact-DV* activate the immune response

**Figure 1:**
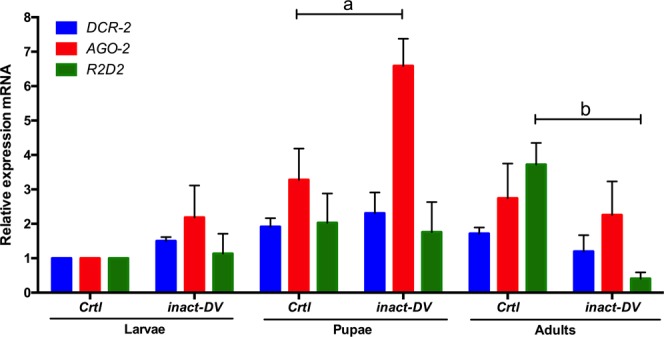
Relative expression of *DCR-2, AGO-2* and *R2D2* in larvae, pupae and adult mosquitoes after exposed with *inact-DV* (UV) in larvae stage for 24 hours. *Crtl* group was exposed only with sterile lap water. Letters above the graph (a and b) indicate statistical differences (*p* < 0.05) in the mean of relative expression between treatments according to *Multiple pairwise t-tests* with Holm correction for multiple testing. The relative expression that is not significantly different between treatments has not a letter. Values are expressed as the mean ± SE. The data presented represents at least three experimental replicates.

**Figure 2:**
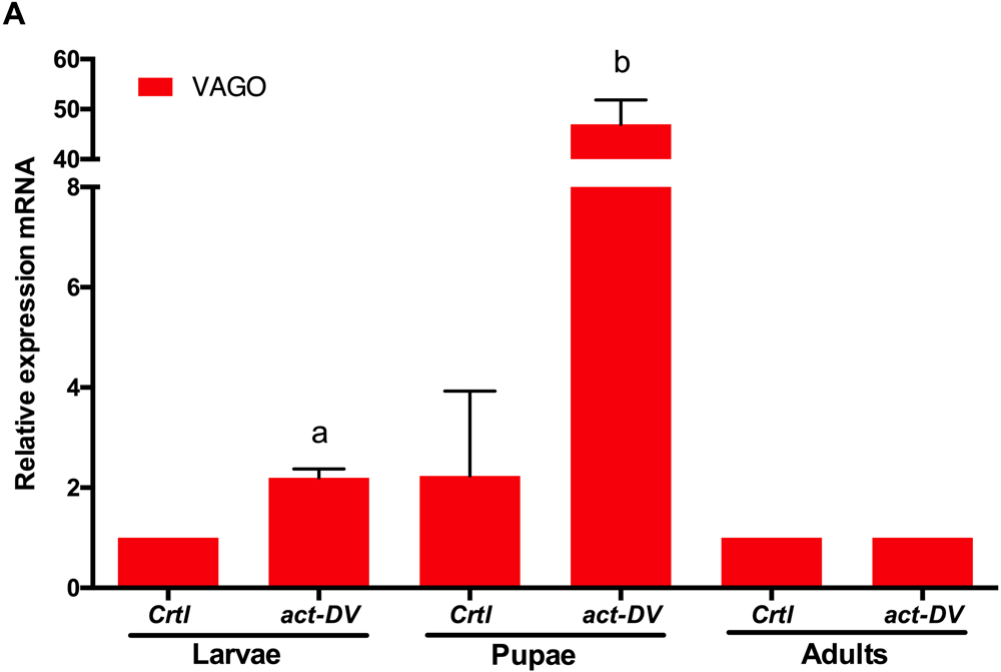
Relative expression of *VAGO* in larvae, pupae, and adult mosquitoes after exposed with *DV* (2.1 × 10^7^ PFU/ml) in the larvae stage for 24 hours. Letters above the graph indicate statistical differences (*p* < 0.05) in the mean of relative expression between treatments according to *Multiple pairwise t-tests* with Holm correction for multiple testing. The relative expression that is not significantly different between treatments has not a letter. Values are expressed as the mean ± SE. The data presented represents at least two experimental replicates.

To confirm non-infection in larvae exposed to *inact-DV* we previously quantified the number of infectious viral particles via a plaque-forming assay (see Supplementary Fig. S4). As expected, 3^rd^ instar larvae, pupae, and adult mosquitoes exposed to *inact-DV* did not show infection (evaluated as a viral load; see Supplementary Fig. S5). Also, the relative expression of *siRNAs* members *AGO-2*, *DCR-2*, and *R2D2* were evaluated as antiviral marker presence in larvae, pupae, and adult mosquitoes after the 1^st^ challenge with *inact-DV*, by real-time PCR (Fig. 1). *AGO-2* was significantly overexpressed in the pupa stage after exposure to *inact-DV*, compared to the control (*Crtl*) group (*p* < 0.05); indicating that *siRNAs* mechanisms were activated. *DCR-2* also showed expression in primed larvae and pupae, but no significant difference was detected to control treatments (*p* = 0.333). However, *R2D2* gene expression was significantly decreased in the adult stage after immune priming with *inact-DV*, in comparison to the *Crtl* group (*p* < 0.05). We also observed significant overexpression of *VAGO* (an interferon-like factor) in larvae and pupae exposed to *act-DV* (*p* < 0.05; Fig. 2).

### Enhancement of the antiviral immune response against dengue virus after the second challenge with active virus

**Figure 3:**
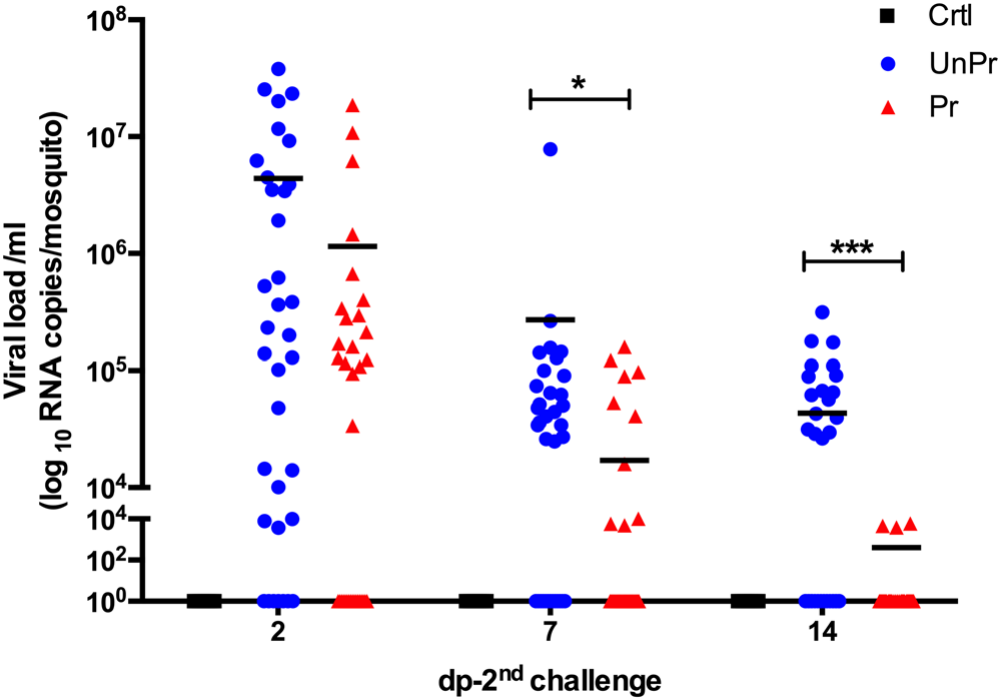
Inter-variation of viral DENV load detected in excreta individual mosquitoes, at 2, 7 and 14 dp-2^nd^ challenge. Blue circle represent DENV RNA copies of unprimed group (*UnPr; N* = 27) were adult mosquitoes that in larvae stage were not exposed with *inact-DV*, and an adult stage were infected with *DV*; red triangle represents individual primed mosquitoes (*Pr; N* = 29) were in larvae stage were exposed with *inact-DV* for 24 hours and in adult stage were fed with *DV* for 1 hour, and black square represents adult mosquitoes that in larvae stage were not exposed with *inact-DV* and in adult stage were fed with rabbit blood (*Crtl; N* = 30). *P*-values represent the statistical significance based on *Mann–Whitney* U-*test*. (*p < 0.05; ***p < 0.001). Values are expressed as the mean. The data presented represents at least three experimental replicates.

The variation in infection between different mosquitoes exposed to DENV, observed in excretes at 2, 7, and 14 days post-2^nd^ challenge (dp-2^nd^ challenge) is shown in Fig. 3. At two dp-2^nd^ challenge, the *UnPr* viral load ranged between 3.7 × 10^3^ and 3.79 × 10^7^ RNA copies per female), and in the *Pr* group, it ranged from 9.4 ×10^4^ to 1.86 × 10^7^ RNA copies per female. These groups showed no significant differences in both treatments (*Mann-Whitney test*, *p* = 0.06). Also, we observed 80% and 71.4% of prevalence for *UnPr* and *Pr* group, respectively (*df* = 1, *X*^2^ = 0.6992, *p* = *0.45;* see Supplementary Fig. S6). At seven dp-2^nd^ challenge, the viral RNA in excretes decreased in both treatments (*UnPr:* 2.4 × 10^4^–7.8 × 10^6^ and *Pr:* 4.8 × 10^4^–1.5 × 10^5^ RNA copies per female); and there were significant differences between groups (*Mann-Whitney test*, *p* < 0.05). Moreover, from a total of 35 females for each treatment, the *UnPr* group had 62.8% of DENV RNA positive excreta, while only 35% of the *Pr* group showed viral RNA (*df* = 1, *X*^2^ = 8.2894, *p* < *0.0*1). This decrease in viral load was also observed in primed females (*Pr*) at 14 dp-2^nd^ challenge. Viral load significantly decreased in individual mosquitoes that were exposed to *inact-DV* during the larval stage, showing viral RNA levels between 3.7 × 10^3^ to 5.8 × 10^3^ RNA copies per female, with an infection percentage of 8.5%. Adult mosquitoes that were not exposed to *inact-DV* in the larval stage (*UnPr* group) showed a viral load between 2.6 × 10^4^–1.7 × 10^5^ RNA copies per female (*Mann-Whitney* test, *p* < 0.001) with a 48.5% infection rate (*df* = *1*, *X*^2^ = 41.86, *p* < 0.001). No viral RNA from DENV was detected in the control (*Crtl)* group at 2, 7, and 14 dp-2^nd^ challenge.

**Figure 4:**
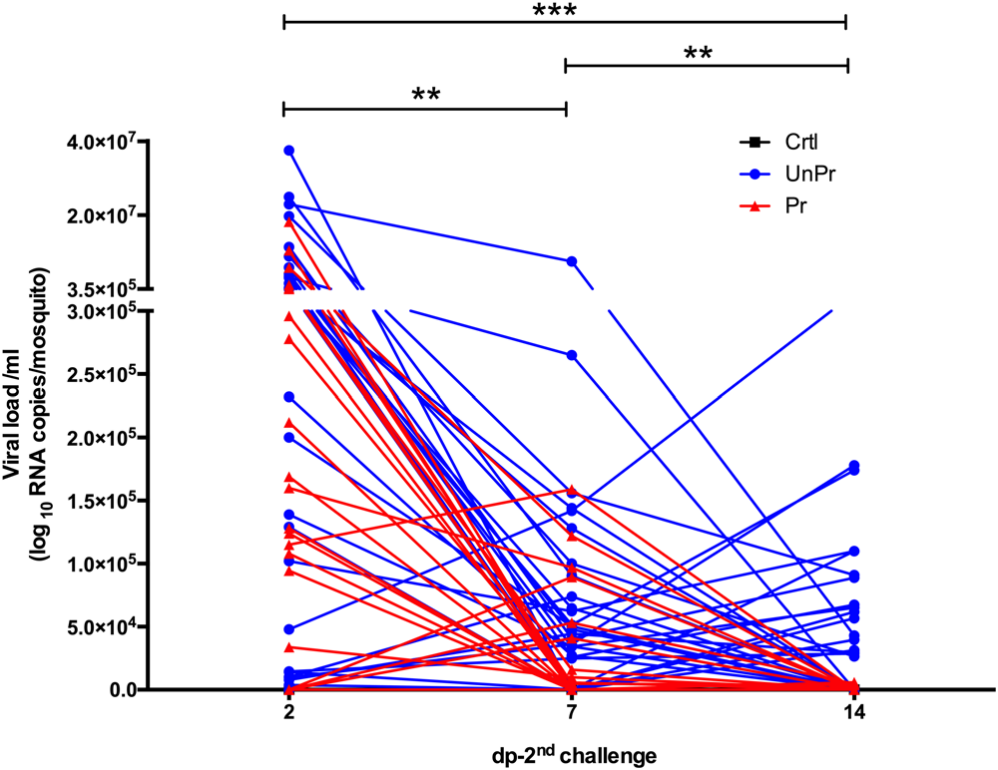
Time course of dynamic of DENV infection of each excreta individual mosquitoes, at 2, 7 and 14 dp-2^nd^ challenge. Blueline represents viral RNA copies of unprimed group (*UnPr; N* = 27) were adult mosquitoes that in larvae stage were not exposed with *inact-DV*, and on adult stage were infected with *DV*; red line represents primed group (*Pr; N* = 29) were adult mosquitoes that in larvae stage were exposed with *inact-DV* for 24 hours and in adult stage were fed with *DV* for 1 hour and black line were adult mosquitoes that in larvae stage were not exposed with *inact-DV* and in adult stage were fed with rabbit blood (*Crtl; N* = 30) over time. *P*-values represent the statistical significance based on *2way ANOVA RM test*. (**p < 0.01; ***p < 0.001). The data presented represents at least three experimental replicates.

**Figure 5:**
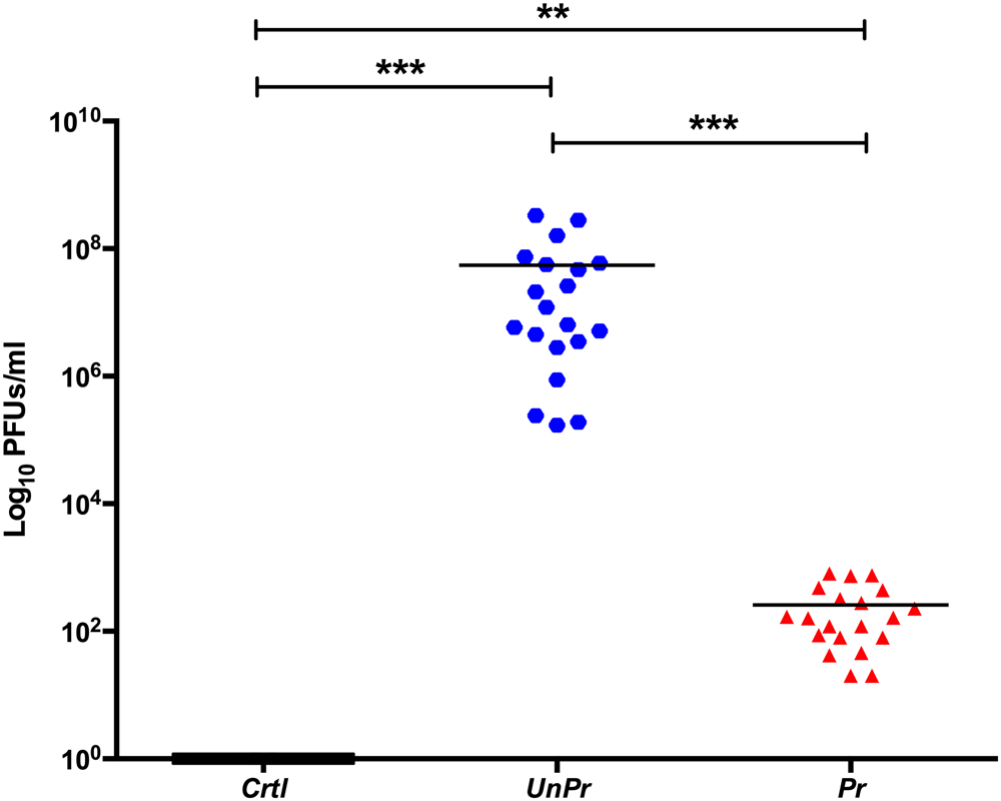
Infections particle count in head individual mosquitoes at 21 dp-2^nd^ challenge for each treatment. Blue circle represent unprimed mosquitoes (*UnPr; N* = 20) were adult mosquitoes that in larvae stage were not exposed with *inact-DV*, and on adult stage were infected with *DV*; red triangle represents primed mosquitoes (*Pr; N* = 20) were adult mosquitoes that in larvae stage were exposed with *inact-DV* for 24 hours and in adult stage were fed with *DV* for 1 hour and black square represent adult mosquitoes that in larvae stage were not exposed with *inact-DV* and in adult stage were fed with rabbit blood (*Crtl; N* = 20). *P*-values represent the statistical significance based on *Mann–Whitney* U-*test*. (**p < 0.01; ***p < 0.001). The data presented represents at least three experimental replicates.

The dynamic disease analysis of DENV infection in individual mosquitoes, on different periods (2, 7, and 14 dp-2^nd^ challenge from each treatment), was carried out without sacrificing mosquitoes (see material and methods). We observed temporal and inter-individual variations among infected mosquitoes (Fig. 4). *Pr* mosquitoes showed a significant decrease in viral RNA load over time, compared to *UnPr* mosquitoes (*Multiple t-tests*; *p* < 0.01), indicating that immune priming with *inact-DV* in the larval stage confers long-lasting protection against DENV in adult mosquitoes (Fig. 4). A decrease of viral RNA load in primed mosquitoes (determined in the excretes) was confirmed by quantifying the plaque-forming units (PFU/ml) on individual heads of adult mosquitoes at 21 dp-2^nd^ challenge (see Supplementary Fig. S7). A significant decrease of viral replication of DENV was observed (Fig. 5), as well as a reduction of viral infective particles in primed adult mosquitoes (*Pr*), compared with mosquitoes that were not primed in the larval stage (*UnPr*; *Mann-Whitney test*, *p* < 0.001).

### Analysis of siRNAs markers in adults after immune priming in the larval stage indicates an improvement in the antiviral immune response against dengue virus

**Figure 6:**
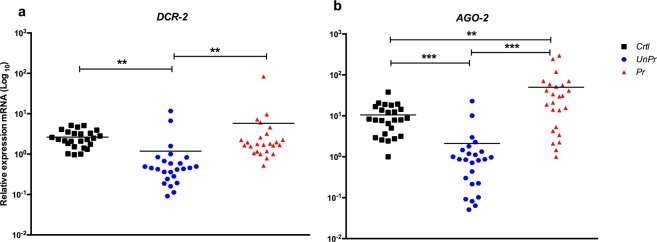
Effect of immune priming on the relative expression of (**a**) *DCR-2* and (**b**) *AGO-2* in abdomen individual mosquitoes at 21 dp-2^nd^ challenge. Blue circle represent unprimed mosquitoes (*UnPr; N* = 25) were adult mosquitoes that in larvae stage were not exposed with *inact-DV*, and on adult stage were infected with *DV*; red triangle represents primed mosquitoes (*Pr; N* = 25) were adult mosquitoes that in larvae stage were exposed with *inact-DV* for 24 hours and in adult stage were fed with *DV* for 1 hour and black square were adult mosquitoes that in larvae stage were not exposed with *inact-DV* and in adult stage were fed with rabbit blood (*Crtl; N* = 25). *P*-values represent the statistical significance based on *Wilcoxon test* (**p < 0.01; ***p < 0.001). Values are expressed as the mean. The data presented represents at least three experimental replicates.

Immune priming with *inact-DV* in the larval stage increased the expression of RNAi components *AGO-2* and *DCR-2* in adult mosquitoes after 21 dp-2^nd^ challenge with DENV (Fig. 6). *AGO-2* and *DCR-2* showed a significant increase in mRNA level expression in primed mosquitoes (*Pr*) compared to the *UnPr* group (*Wilcoxon test*, *p* < 0.001 and *p* < 0.01, respectively; Fig. 6A,B). However, no significant difference in *DCR-2* expression was observed between *Crtl* and *Pr* mosquitoes (*Wilcoxon test*, *p* = 0.423; Fig. 6A); while *AGO-2* showed a significant difference in relative expression between the *Crtl* and *Pr* groups (*Wilcoxon test, p* < 0.001; Fig. 6B). Otherwise, *DCR-2* and *AGO-2* showed a significant decrease in mRNA expression levels in unprimed mosquitoes (*UnPr*) compared to the *Crtl* group (*Wilcoxon test, p* < 0.0001 and *p* < 0.001, respectively; Fig. 6A,B).

## Discussion

**Figure 7:**
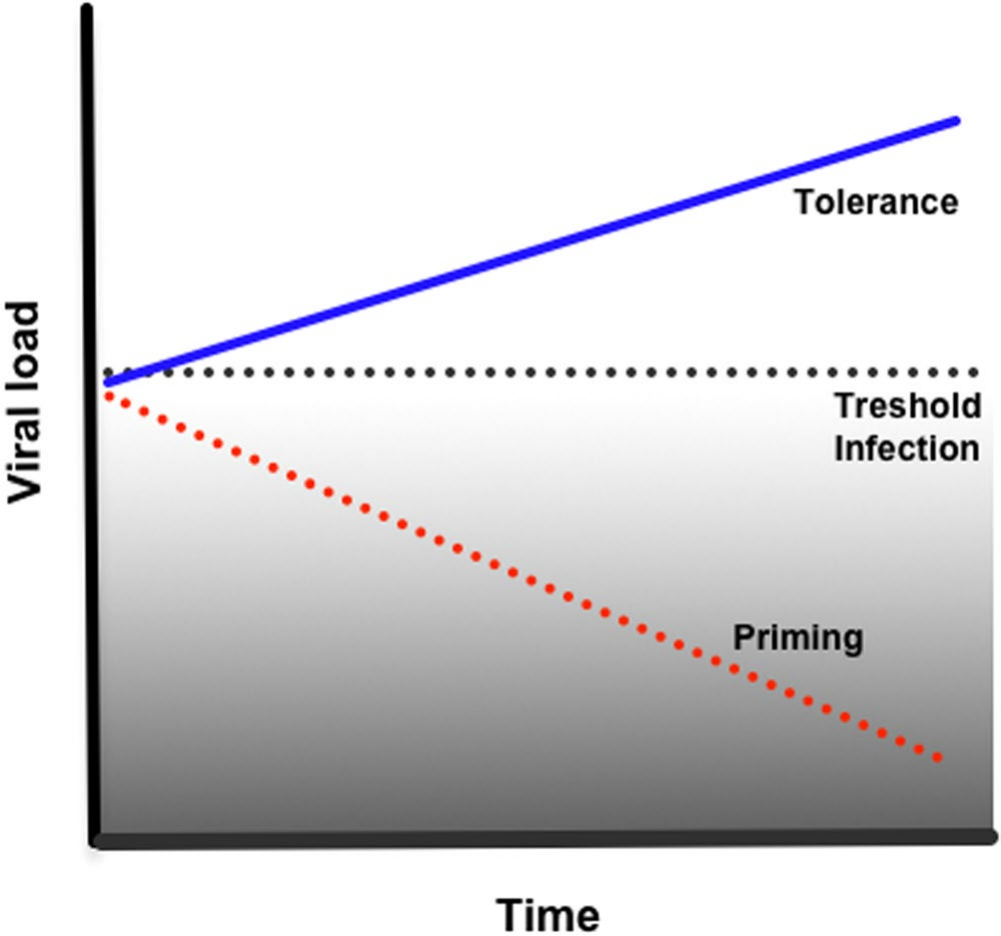
Priming and tolerance curve in mosquitoes infected with DENV. It is known that mosquitoes infected with dengue virus, maintain an infection level below a threshold of damage, but high enough to pass the infection threshold, keeping mosquitoes in a condition of tolerance promoting the arbovirus transmission cycle. When we boost the immune response to a first viral challenge in the ontogeny of vector mosquitoes, we observe a decrease in viral load in newly infected adult mosquitoes, because, over time, we find individual mosquitoes with a reduction in viral load concerning control. We believe that priming breaks the immune tolerance, lowering the viral load to lower levels of the infection threshold.

Immune priming with *inact-DV* in the larval stage protects against *act-DV* infection in adult mosquitoes. Adult mosquitoes coming from primed larvae with *inact-DV* showed reduced levels of virus particles in the head, compared to adults coming from unprimed larvae. We suggest that immune priming at the larval stage with *inact-DV*, confers a virus resistance condition to *act-DV* in the adult stage, breaking the tolerance, and the siRNAs pathway likely participates in the antiviral immune response, without ruling out that other immune pathways might be involved. There are few studies in insects where immune priming in a larval instar protects the adult stage. We have documented a protective effect in adult *Ae. aegypti* mosquitoes which resist a killing dose of bacteria if the 4^th^-larval stage is previously primed with *Escherichia coli*^9^. Tidbury *et al*.^13^ determined that 8 day-old *Plodia interpunctella* larvae, primed with a low dose of *P. interpunctella* granulosis virus, could survive a lethal virus challenge in the adult stage. Likewise, Brown *et al*.^16^ observed that *Anopheles gambie* adult mosquitoes from infected larvae have stronger antibacterial activity in their hemocoel. They also showed increased susceptibility to malaria infection. As far as know, our results are the first evidence that immune priming from larval stages might reduce the tolerance or susceptibility condition to a viral infection in adult mosquitoes. It has been proposed that decreasing mosquito tolerance to arbovirus infection could be a successful disease control strategy than increasing mosquito resistance. Reducing tolerance should lead to the elimination or reduction in virus load, making mosquitoes less susceptible to transmit dengue^17^. These observations and our results indicate that insects can display protection against different pathogens if primed during the larval stage (Fig. 7); however, the mechanisms behind this protection require further research.

During DENV infection, the mosquito immune response is activated. Four fundamental signaling pathways: RNA interference (RNAi), Toll, IMD, and JAK-STAT participate in controlling the infection. RNAi has been proposed as the most critical antiviral response in all arthropods^24^. There are three major types of RNAi systems: the microRNA (*miRNA*), PIWI-interacting RNA (*piRNA*), and small interfering RNA (*siRNAs*) pathways. The *siRNAs* pathway is considered to be the most crucial antiviral immune response in arbovirus vectors, such as mosquitoes^24,25^. Upon virus infection, RNA viruses generate foreign dsRNA in the insect’s cell as replication intermediates; the dsRNA is recognized as a pathogen-associated molecular pattern (PAMP) by *DCR-2* and in association with *R2D2*, *DCR-2* cleaves dsRNA into virus-induced *siRNAs*, designated *viRNAs* (when are derived from virus), usually 21–24 nucleotide long. The *viRNAs* are then incorporated into the RNA-induced silencing complex (*RISC*) with *AGO-2* as its integral protein^20,21,24^. *AGO-2* is also called the slicer protein, which is considered essential for an efficient RNAi response against flaviviruses^26^.

In this study, we observed the overexpression of *AGO-2* in the pupae stage after the 1^st^ challenge (priming) with *inact-DV* in larvae, suggesting that the *siRNAs* mechanism was activated, even though there was not *act-DV*. *AGO-2* expression in *Ae. aegypti* pupae after contact with a non-infective virus, indicate that pupae instar stages can express antiviral genes. Maybe, the inactivation of DENV by UV induces the dimerization of proximal pyrimidines from a single DENV RNA strand (*dimer RNA*+), which generates *dsRNA* from the virus^27^. We consider that when *Ae. aegypti* larvae were exposed to *inact-DV*, some RNA dimers might activate the direct expression of *AGO-2* in the pupal stage and loaded into the Argonaute-containing effector complex (*RISC*; the RNA-induced silencing complex). One of the strands of *viRNA* is cleared and degraded by *AGO-2*. We propose that the activation of *siRNAs* in the larval stage with *inact-DV* might enhance the antiviral immune response in the adult stage.

Interestingly, *DCR-2* and *R2D2* expression in larvae, pupae, and adult mosquitoes after the first challenge was not modified in relation to the control treatment. Probably, the mRNA level of these genes is not modified because larvae were exposed to *inact-DV*, and their expression requires *act-DV* (see Supplementary Figs. S8 and S9). However, the previous exposure to *inact-DV* in *Ae. aegypti* larvae enhance the antiviral immune response against DENV in adult mosquitoes, modifying the expression of *DCR-2* and *AGO-2* after viral infection (see Fig. 6). This enhancement in the immune system of adult mosquitoes was induced with immune priming in larvae without any visible damage or infection caused by DENV. Therefore, we suggest that the recognition of infectious viral particles is not required to induce immune priming in the larval stage. When adult mosquitoes were infected with *act-DV*, likely the *siRNAs* were already overexpressed, as well as *AGO-2* and *DCR-2*, to limit DENV replication. Nevertheless, *siRNAs* are not exclusive for viral clearance, and other immune mechanisms could act during oral infections, as was reported in *Drosophila* infected with different viruses^15^. Currently, our knowledge about the antiviral immune response in insects remains limited^20,28–31^.

Otherwise, either with or without immune priming, *VAGO* is not expressed in adult mosquitoes. However, an interesting observation was the expression of *VAGO* in the pupal stage (Fig. 2). The expression of *VAGO* has been reported in *Culex* mosquitoes infected with West Nile virus^22^, in RML12 cells infected with DENV^23^ and in *Ae. aegypti* mosquitoes infected with *Wolbachia*^32^. To our knowledge, this is the first observation of *VAGO* overexpression in the pupa stage due to DENV contact; opening the possibility that *VAGO* has a critical antiviral function during ontogeny. Further research is required to determine the role of this molecule and the RNAi system in the control of viral infections in the mosquito aquatic phases.

The mechanisms behind immune priming induced from the larval stage have not been ascertained yet. Nevertheless, we have recently observed that DNA synthesis and endoreplication participates in the immune priming of adult mosquitoes against DENV and *Plasmodium*^6,7^. Immune priming in the *Anopheles albimanus* LSB-AA695BB cell line induced DNA synthesis, increasing the number of *TEP* and *PPO1* gene-copies^7^. It is noteworthy that endoreplication may also participate during immune priming in the larval stage, probably making copies of antiviral genes like *AGO-2*. Copies of the *AGO-2* gene can be transcribed rapidly after the second challenge. This consideration requires further investigation.

Intervariation in DENV infection has been reported in individual *Ae. aegypti* adult mosquitoes^19,33^; however, this evaluation needs all mosquito tissues to be processed, so individuals must be sacrificed. In our study, the dynamic of DENV infection in the same mosquito species was evaluated at different time-periods. This procedure allowed us to study mosquitoes of the same strain having a different infection dynamic (some mosquitoes became infected early, others late or even resolved the infection over time). It would be interesting to identify the immune genes and molecules in individuals that showed different capabilities to control the viral infection, to determine the specific immune molecules that may control virus replication with and without priming.

In conclusion, the induction of immune priming in *Ae. aegypti* larvae open novel targets to induce DENV transmission resistance, modifying the immune response from this aquatic life stage.

## Methods

### Breeding of larvae and adult mosquitoes

*Ae. aegypti* Rockefeller strain was reared in the insectary of Instituto Nacional de Salud Publica (*INSP*), Mexico. Mosquitoes were maintained under controlled insectary conditions: 27–29 °C, 60–80% relative humidity and a 12:12 h light/dark cycle. Larvae were fed with a standard mix diet of dechlorinated tap water with yeast extract, lactalbumin and mouse pellets in a 1:1:1 proportion, and were reared in plastic trays at a density of about 200 larvae per tray until pupation. Pupae were transferred into cages for emergence. Adult females were maintained with a 10% sucrose solution up until 12 hours before infection; at 3–5 days post-emergence were fed with rabbit blood.

### Experimental infections

The experimental design consists of two processes (see Supplementary Fig. S1): immune priming or first challenge (1^st^ challenge) was done in 3^rd^ instar larvae, and the second challenge (2^nd^ challenge) was carried out in adult female mosquitoes (3–5 days post-emergence). This study was approved by the Biosafety and Ethics Committees of the Instituto Nacional de Salud Pública (INSP, Mexico). We confirm that all experiments were performed in accordance with relevant guidelines and regulations.

### Immune priming in larvae

During the 1^st^ challenge, 600 larvae were separated into two groups: i) 400 larvae were kept in 400 ml of sterile tap water and ii) 200 larvae were primed with 2 ml *inact-DV* (titrated DENV-4 infected LLC-MK2 cell lysate, exposed to UV-light for 1 h at 4 °C) in 198 ml of sterile tap water (1:100). Both treatments were incubated for 24 hours at insectary controlled conditions. Afterward, the larvae were washed three times with tap water and were placed in containers with 2 liters of tap water, where they were left until pupae and adults emerged.

The relative expression of immune response genes was evaluated to establish an effective exposure solution for larvae. For this purpose, a serial *act-DV* dilutions assay (10^6^–10^1^; see Supplementary Fig. S2) and different vehicle mediums were evaluated (MEM, PBS 1×, 0.1% saline solution and sterile tap water; see Supplementary Fig. S3). To confirm non-infection in larvae treated with *inact-DV*, the viral load was analyzed in larvae, pupae, and adult mosquitoes post-1^st^ challenge (see Supplementary Fig. S5).

### Adult mosquito second challenge

In the 2^nd^ challenge (see Supplementary Fig. S1), one group of 100 female mosquitoes (exposed to sterile tap water in their larval stage) were fed with rabbit blood (*Crtl*) and the second group of 100 adults were infected with a 1:1 mixture of *act-DV* and rabbit blood (*UnPr*; one volume of rabbit erythrocytes and one volume of 2.1 × 10^7^ PFU/ml viral titration). A third group consisted of 100 mosquitoes that were primed as larvae with *inact-DV;* these individuals were infected with a 1:1 *act-DV* in rabbit blood (*Pr*) mixture. During infections, blood was maintained at 37 °C for 1 hour. To quantify the PFU/ml used for mosquito infection, the infected blood was collected and stored at -80 °C for later titration as described above. After the 2^nd^ challenge, blood-filled females were selected and transferred individually to small plastic containers (100-mm high, 70-mm diameter) and maintained at insectary controlled conditions. A filter paper disk was placed below each plastic container, and on top of the container was placed cotton soaked in a colored honey solution. The colored honey solution consisted of 5 g of bee honey diluted in 100 μl of blue food dye (*Deiman, Mexico*) and 10 ml of sterile tap water. This solution allowed the visualizing of colored excreta spots on the filter paper (see Supplementary Fig. S10). This method allows the monitoring of viral dissemination in individual mosquitoes without sacrificing them^19^. Kinetic infection of RNA virus in individual excreta was detected at 2, 7, 14, and 21 days post-infection (dpi; see Supplementary Fig. S11).

### DENV propagation and titration

We used DENV serotype 4 (kindly donated by Dr. Rosa Ma. Del Angel, CINVESTAV, IPN). Virus propagation was carried out using Aag2 cells, which were grown at 28 °C in supplemented Schneider’s Drosophila medium (*Invitrogen*). We use non-infected Aag2 cells as mock cells. Virus titration was performed via a plaque-forming assay before experimental infection, using LLC-MK2 cells. Confluent monolayers of LLC-MK2 cells in a 24-well sterile plate were infected with 0.1 ml of inoculum. Afterward, a 10-fold dilution series was generated (10^−3^ to 10^−4^) and incubated for one h at 37 °C and 5% CO_2_. Each well was supplemented with 500 μl of minimal essential medium (MEM), 500 μl of 2.5% methylcellulose and incubated for seven days at 37 °C and 5% CO_2_, until cytopathic effects were observed. The viral titration was determined by counting directly the number of lytic plates found at the highest dilution. The next Eq. (1) was employed considering the dilution factor; where PFU is the plate-forming unit, df is the dilution factor, and 10 is the correction factor corresponding to 0.1 ml of the viral inoculum.

1 $$PFU/ml=(observed\,number\,of\,plates)\ast (10)\ast (df)$$

### Viral RNA detection in mosquito excreta

Infection kinetics analysis was performed at 2, 7, and 14 days post-2^nd^ challenge (dp-2^nd^ challenge), to study the dynamics of infection without sacrificing adult mosquitoes (see Supplementary Fig. S10). For this, a protocol modified by Fontaine *et al*.^19^, was used to detect the viral load in mosquito excreta. Excreta spots per mosquito were collected on filter paper using a cutting mat (*Harris, Uni-Core*). The spots were resuspended in 200 μl PBS 1X in a 1.5 ml microtube, 100 μl of viral lysis buffer were added to the samples (*Qiagen*) and incubated for 20 min at 56 °C. After vortexing for 20 sec, samples were centrifuged for 1 min at 8,000 rpm, and 300 μl of the solution was recovered. Total RNA from individual excreta was extracted using a *Qiamp Viral RNA mini kit* (*Qiagen*), according to the manufacturer’s instructions. At the final step, total RNA from each sample was eluted in 60 μl of RNase-free water, and RNA concentration was measured using a NanoDrop Lite (*Thermo Scientific*). We normalized at 50 ng/μl of the total RNA for cDNA synthesis. We used *RevertAid Premium Reverse Transcriptase* (*Thermo Scientific*) and 1 μl of cDNA for real-time quantitative PCR reactions; using *Maxima SYBR Green/ROX qPCR Master Mix* (*Thermo Scientific*) on a *ViiA 7 Real-Time PCR System* (*Thermo Scientific)*. For absolute quantification, a standard curve was generated using a 10-fold serial dilution of a DENV insert-containing plasmid, of known concentration number of dengue genome copies (8.38 × 10^7^ Dengue genome copies per microliter, donated by Dr. Rosa Ma. Del Angel, CINVESTAV, IPN). Total viral load per mosquito excreta was extrapolated from *C*_*T*_ values using least-squares fitting (*ViiA 7 Software*). The primers designed to amplify the DENV universal region were DENV (Fw) 5′-CAA TAT GCT GAA ACG CGA GAG AA-3′, DENV (Rv) 5′-CCC CAT CTA TTC AGA ATC CCT GC-3′ for a 200 bp amplicon. The *C*_*T*_ range for a positive sample was between 19.26 to 33.5; more than 33.5 was considered as a negative sample.

### Lytic plaque assay of individual mosquitoes

To confirm the viral RNA load observed in mosquito excretes, we quantify the plaque-forming units (PFU/ml) on individual heads of adult mosquitoes at 21 dp-2^nd^ challenge (see Fig. 5 and Supplementary Fig. S7). Twenty mosquito heads per treatments were dissected and placed in 500 μl of 1X PBS in a 1.5 ml microtube. To each sample, five μl of protease inhibitor cocktail (*Complete, Mini, Roche*) were added and homogenized with biovortex (*Diagger*) for 30 sec. Supernatant samples were filtered with 0.2 μm syringe filters − 13 mm PVDF membrane (*Millex*) and stored at −70 °C. A sub-confluent culture of LLC-MK2 cells was infected with 200 μl of supernatant in 96-well sterile plates (*Corning*). This was done in duplicate using 10-fold serial dilutions of total supernatant. Plates were incubated for one hour at 37 °C in a 5% CO_2_ atmosphere, and 100 μl of minimal essential medium (*MEM*) was added per well. Infected cells were incubated for 4–7 days at 37 °C in 5% CO2 until cytopathic effects were observed. The mosquito heads titer were expressed as plaque-forming units per milliliter (PFU/ml).

### Gene expression analysis by RT-qPCR

Twenty-five individual abdomens were dissected 21 days post-2^nd^ challenge and placed in 500 μl of *Trizol* (*Invitrogen*) in a 1.5 ml microtube. Samples were homogenized for 30 sec, and 200 μl of chloroform (*Sigma-Aldrich*) was added, followed by vortexing for 30 sec. After 5 min incubation at 4 °C, samples were centrifuged at 4 °C for 15 min at 13,500 rpm. The upper aqueous phase was harvested and transferred to a new 1.5 ml microtube containing 500 μl of isopropanol (*Sigma-Aldrich*) and gently mixed for 20 sec. Samples were incubated at −20 °C overnight and centrifuged at 4 °C for 10 min at 13,500 rpm to pellet the RNA. Sample pellets were washed with 500 μl of 70% ice-cold ethanol (*Sigma-Aldrich*) and centrifuged at 4 °C for 10 min at 13, 500 rpm. Samples were allowed to dry for 5 min at 56 °C. Total RNA was resuspended in 30 μl of RNase-free water, and all samples were stored at −80 °C until used. The RNA concentration was measured using a NanoDrop Lite (*Thermo Scientific*). We normalized at 500 ng/μl of the total RNA obtained from individual abdomens to acquire cDNA, using *RevertAid Premium Reverse Transcriptase* (*Thermo Scientific*). Gene expression was assayed by RT-qPCR using a *ViiA 7 Real-Time PCR System* (*Applied Biosystem)*. The qPCR master mix contained 0.4 μM of each primer, 5 μl of 1X *Maxima SYBR Green/ROX* (*Thermo Scientific*), 3 μl of cDNA template and 1.2 μl of RNase-free water for a final 10 μl volume. Specific *primers* for the *siRNAs* pathway were designed as markers for the antiviral immune response after the 2^nd^ challenge. The primers used were: Argonaute-2, *AGO-2*; Fw: 5′-CAG TGC GTT CAG GCC AAA AA-3′; Rv: 5′-TCC ACC CAG TTT GAC GTT GA-3′; Dicer-2, *DCR-2*; Fw: 5′-CGA AGA GGT CAT TGG TGG CT-3′; Rv: 5′-CAC GGC AGA GGT ATA TCG CC-3′; R2D2, *R2D2*; Fw: 5′-CAC TTT TTG GCG GTC CTG TC-3′; Rv: 5′-TTC GGG GCA TCT CGA AGT TC-3′; Vago, *VAGO*; Fw: 5′-TGA CGA GGA GAA ATC CAT CC-3′; Rv: 5′-TCC CAT CGT GTA CGC ATT TA-3′ and ribosomal protein S7, *S7* internal control; Fw: 5′-GGG ACA AAT CGG CCA GGC TAT C-3′; Rv: 5′-TCG TGG ACG CTT CTG CTT GTT G-3′. Relative quantification of mRNA levels was done by the 2^−ΔΔCT^ method, and primer efficiencies were calculated by measuring how the standard ΔC_T_ varied with template serial dilutions (PCR efficiency is about 95–99% for each *primer*). For all assay, the ribosomal protein *S7* gene was used as the reference. The levels of *DEF, CEC, GAM, ATA, REL 1, CAC*, *AGO-2*, *DCR-2, R2D2*, and *VAGO*, were normalized with respect to the *S7* transcript of the same sample. Melting curve analyses confirmed that only cDNA, and not genomic DNA, was amplified. Therefore, we standardized these differences of copy numbers in ratios for all *primers*. Three independent assays were conducted, each analyzed in duplicate.

### Statistical analyses

Data on infection variation of the DENV viral RNA load, in individual excreta, was expressed as the mean and standard deviation, and compared using a *Mann-Whitney U test*. The data of DENV infection dynamics in individual excreta over time were determined by pairwise differences of viral load between time points by *Multiple t-tests* with the *Holm-Sidak* correction for multiple testing. The data analysis for mRNA relative expression was performed by pairwise comparisons using a non-parametric *Wilcoxon test*. All statistical analyses were performed in *Graph Pad Prism* version 6 Oc. (CA, USA).

### Ethical review

This study was approved by the Biosafety and Ethics Committees of the Instituto Nacional de Salud Pública (INSP, Mexico).

## Supplementary information

Supplementary material.
