# Supplementary material for: Immune priming with inactive dengue virus during the larval stage of Aedes aegypti protects against the infection in adult mosquitoes
Source: Sci Rep. 2020 Apr 21;10:6723. doi: 10.1038/s41598-020-63402-z (PMC7174395; doi:10.1038/s41598-020-63402-z)
Supplement: Supplementary file 1 — Supplementary material. [file 41598_2020_63402_MOESM1_ESM.pdf]

Immune priming with inactive dengue virus during the larval stage of *Aedes aegypti* protects against the infection in adult mosquitoes.

Valeria Vargas[1], Jorge Cime-Castillo[1,] & Humberto Lanz-Mendoza[1*]

[1] Centro de Investigaciones sobre Enfermedades Infecciosas, Instituto Nacional de Salud Pública, Cuernavaca, Morelos, Mexico.

[*] Correspondence author. E-mail: humberto@insp.mx

Supplementary Figures

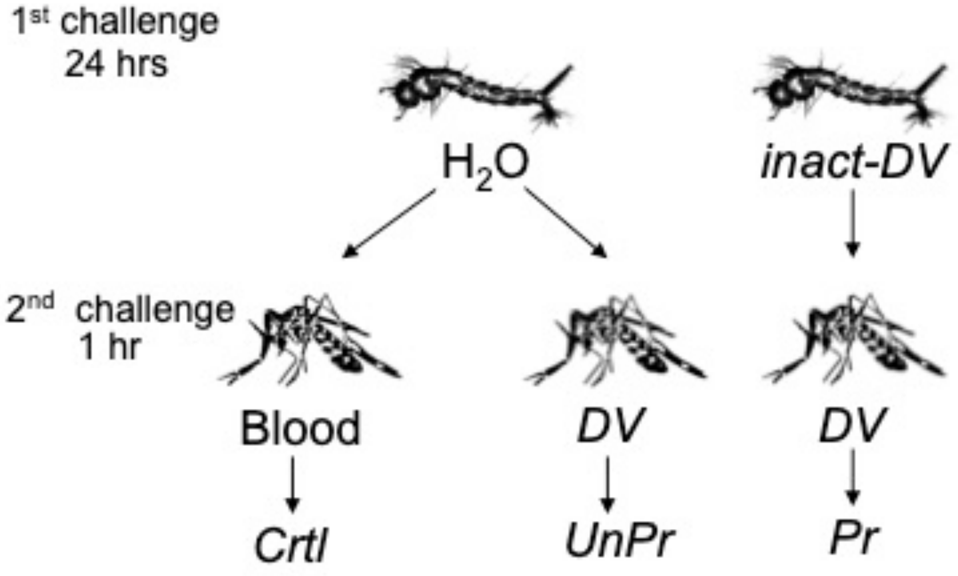

Supplementary Figure 1. Experimental design of immune priming (1st challenge) with inactived-DENV (*inact-DV*) in larvae stage and second challenge (2nd challenge) with active-DENV (*DV*) in adult mosquitoes of *Ae. aegytpi*. Primed mosquitoes (*Pr*) were adult mosquitoes that in larvae stage were exposed with *inact-DV* for 24 hours and in adult stage were fed with *DV* for 1 hour. Unprimed mosquitoes (*UnPr*) were adult mosquitoes that in larvae stage were not exposed with *inact-DV* and in adult stage were infected with *DV*. Control mosquitoes (*Crlt*) were adult mosquitoes that in larvae stage were not exposed with *inact-DV* and in adult stage were fed with rabbit blood.

RIBOSOMAL S7 (S7)

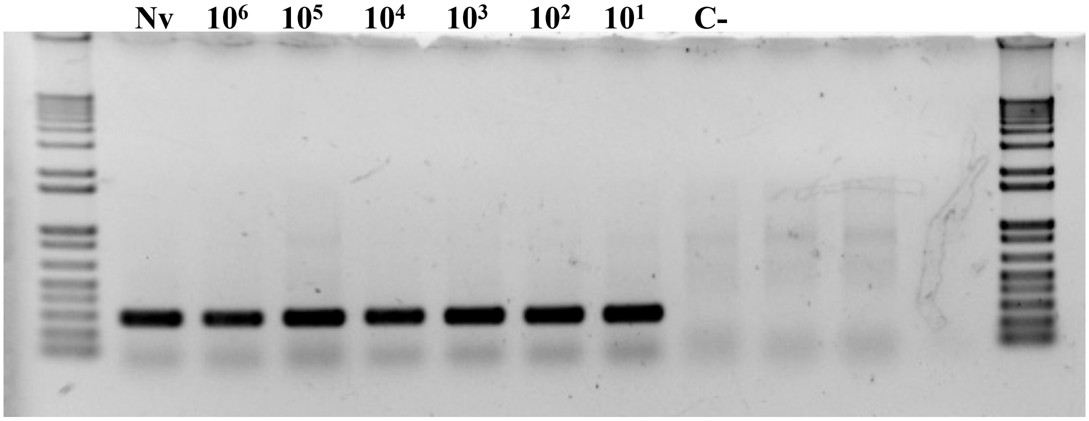

DEFENSIN (DEF)

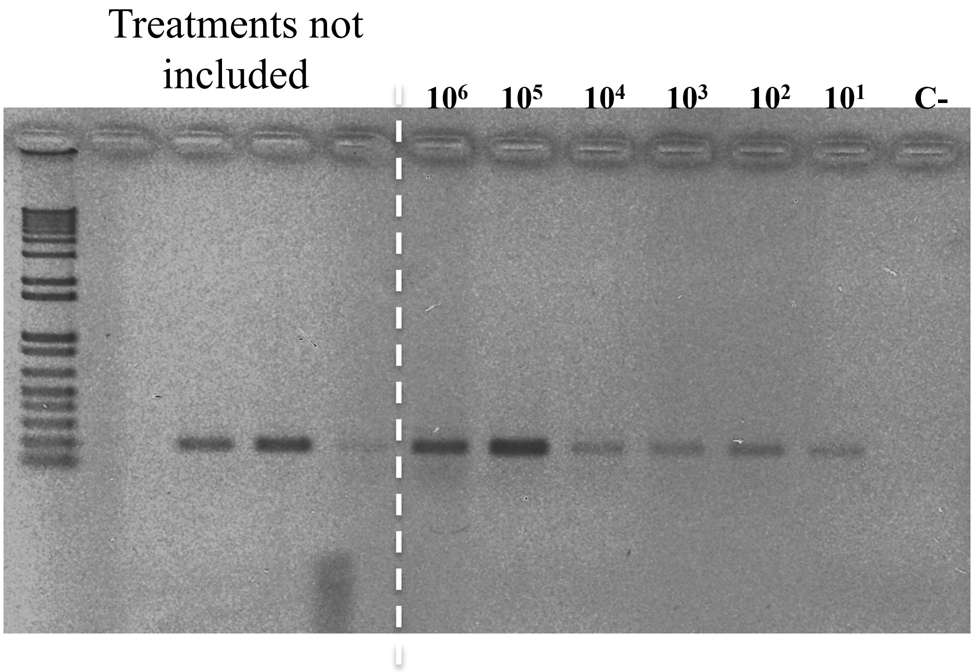

ATTACIN (ATA)

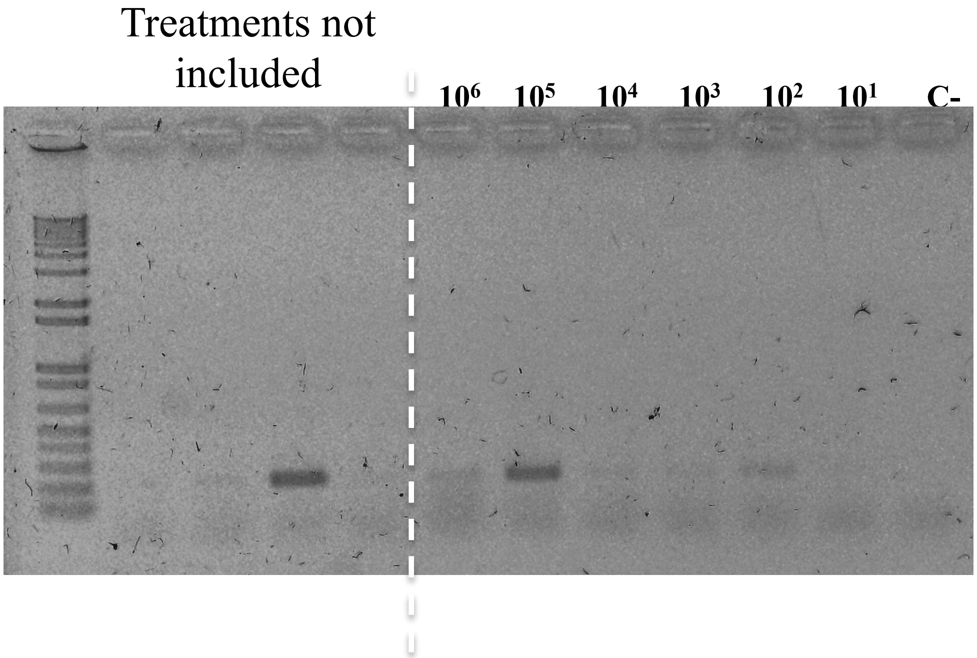

CECROPIN (CEC)

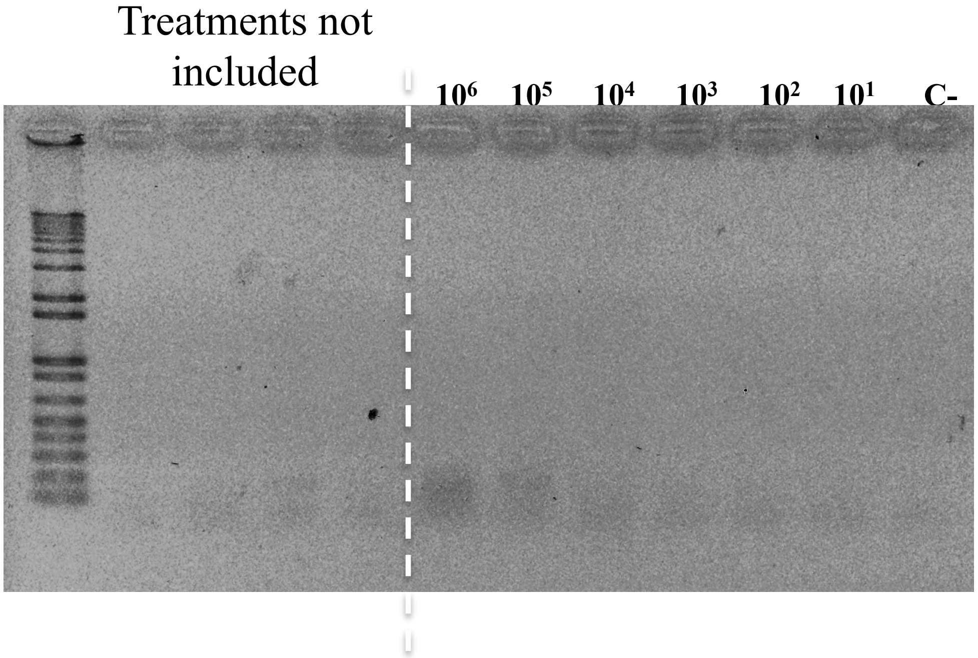

RELISH 1 (REL 1)

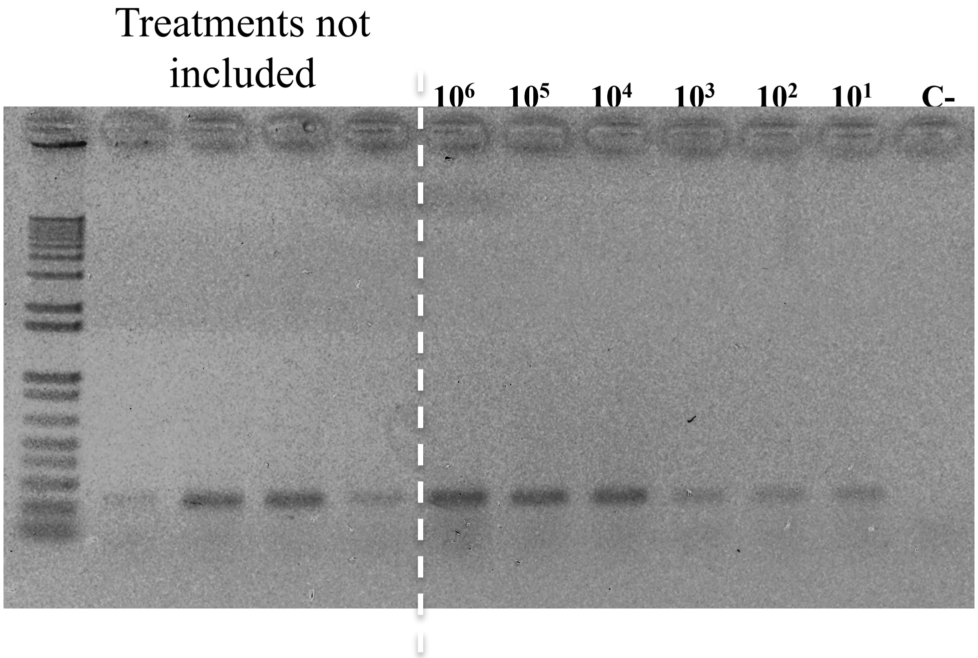

CACTUS (CAC)

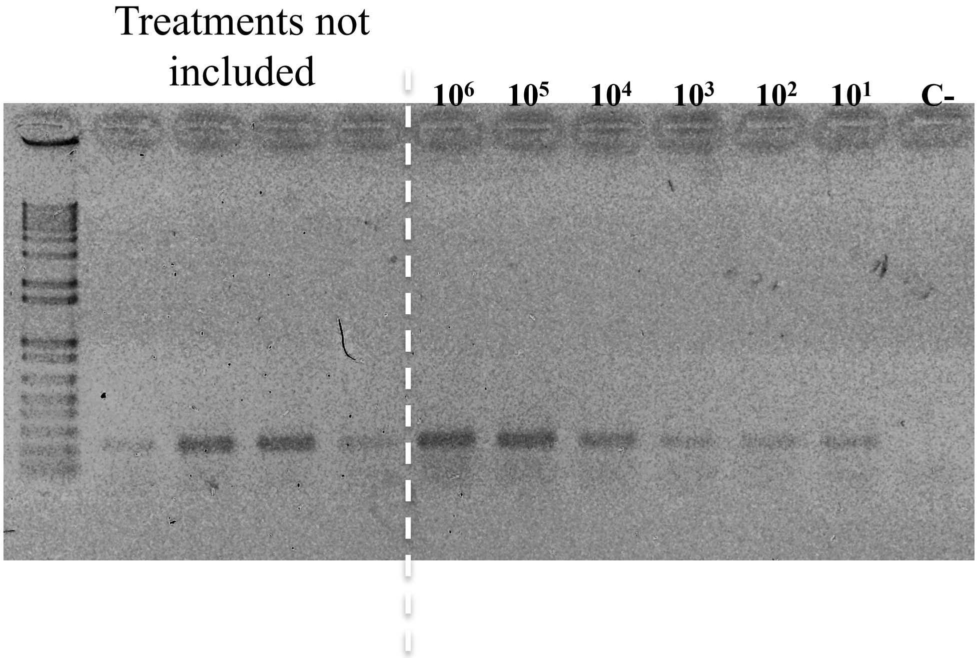

Fig. S2

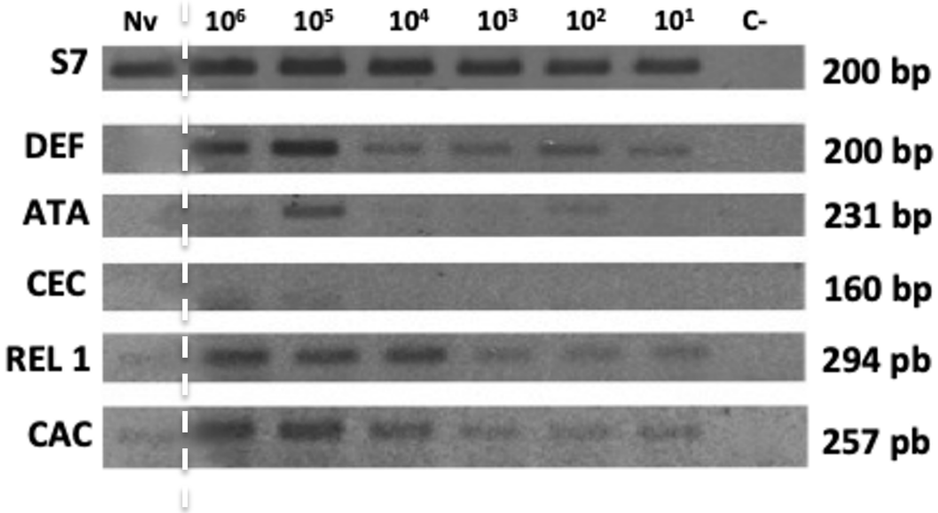

Supplementary Figure 2. Expression mRNA level of Defensin (DEF), Attacin (ATA), Cecropin (CEC), Relish 1 (REL 1) and Cactus (CAC) by RT-PCR in larvae of *Ae. aegypti* at 24 hours post-challenge with 10-fold dilutions assay of *DV* (10^6 - 10^1). Third instar larvae were exposed with dilution of LLC-MK2 cell infected with *act-DV* ($2x10^7$ PFU/ml) in sterile tap water. Gene housekeeping ribosomal S7 (S7). Naïve larvae (Nv) and negative control (C-). N= 10 larvae. The white line represents the court that was carried out for the elimination of a part of the original gel. The data presented represents at least three experimental replicates.

RIBOSOMAL (S7)

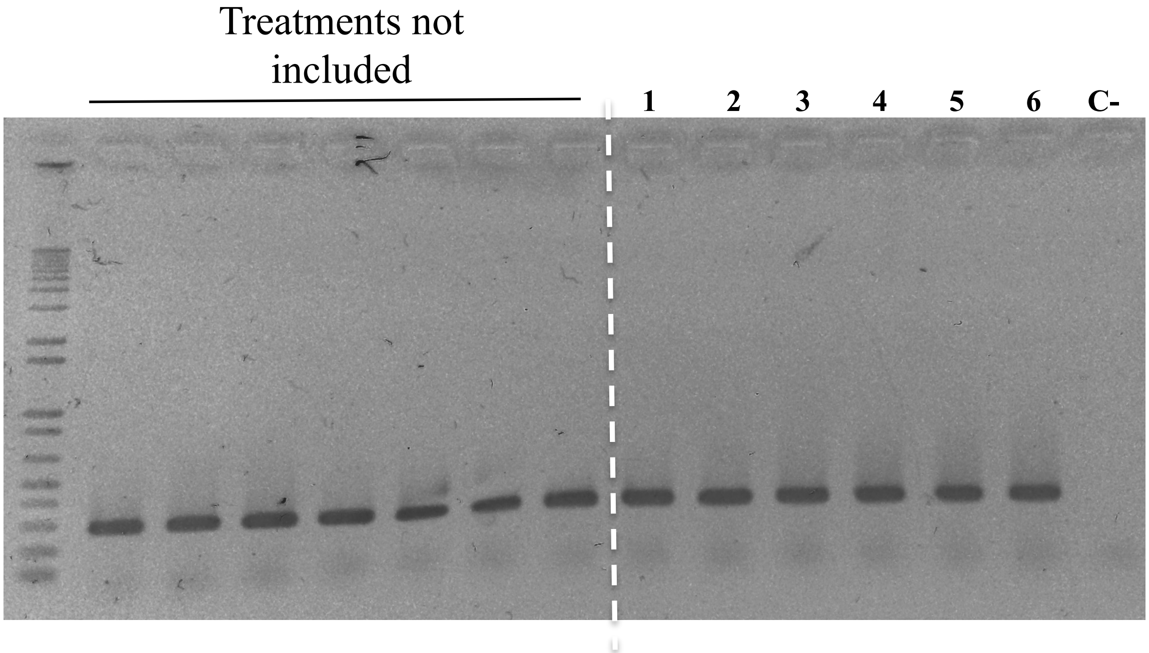

DEFENSIN (DEF)

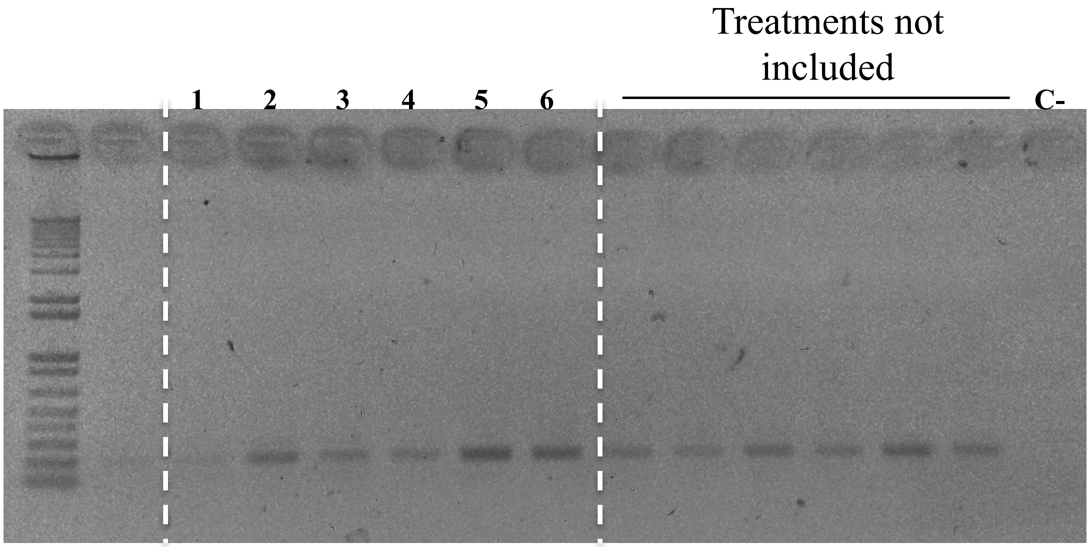

ATTACIN (ATA)

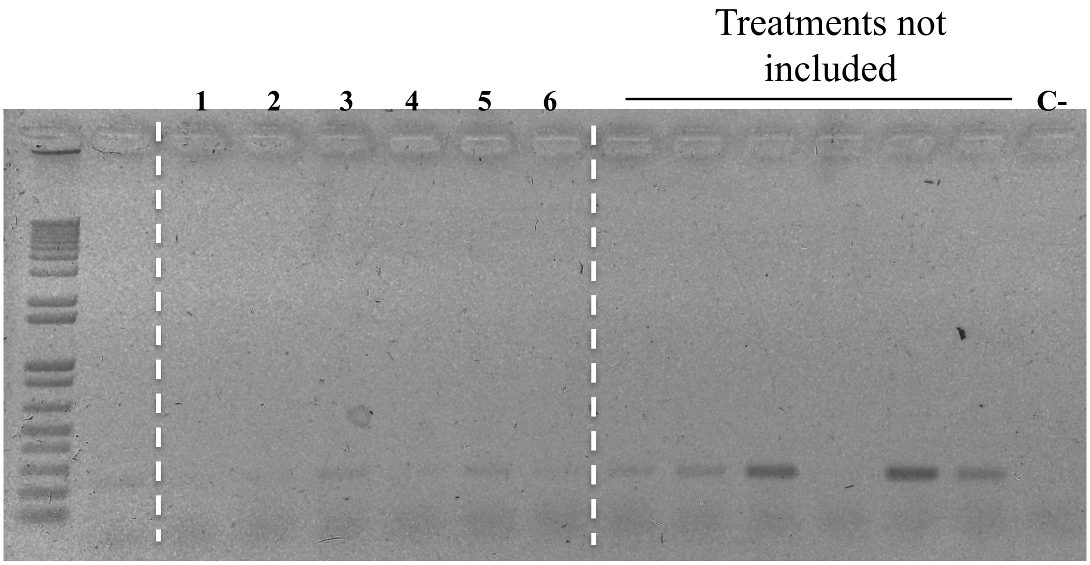

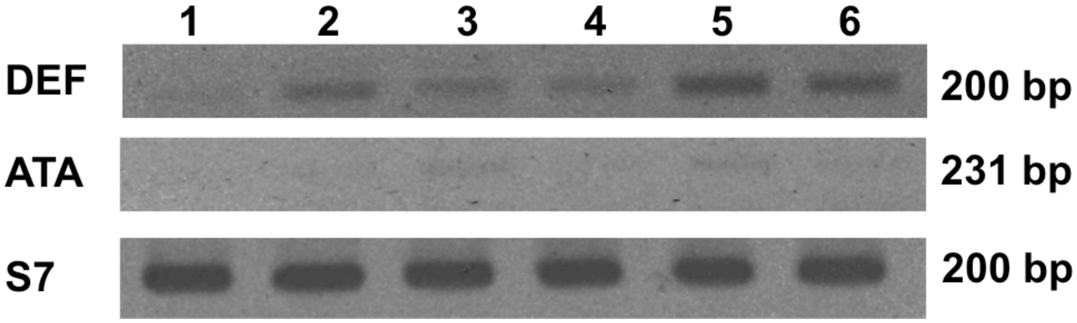

Supplementary Figure 3. Expression mRNA level of Defensin (DEF) and Attacin (ATA) by RT-PCR in larvae of *Ae. aegypti* at 24 hours post-challenge with *act-DV* ($2x10^5$ PFU/ml) in different solutions. Lane 1: Larvae exposed in sterile tap water (H_2O); Lane 2: Larvae exposed in 0.1% saline solution (SS); Lane 3: Larvae exposed in Potassium saline buffer (PBS); Lane 4: Larvae exposed in H_2O with *act-DV*; Lane 5: Larvae exposed in SS with *act-DV* and Lane 6: Larvae exposed in PBS with *act-DV*. N= 10 larvae. The white line represents the court that was carried out for the elimination of a part of the original gel. The data presented represents at least three experimental replicates.

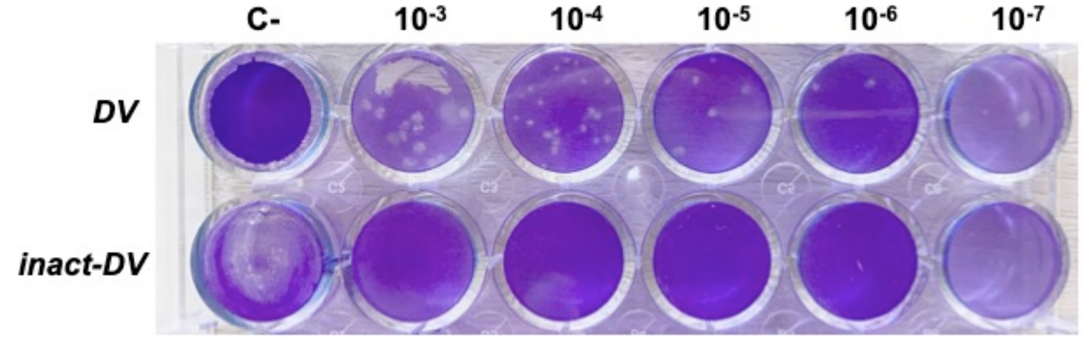

Supplementary Figure 4. Confirmation of inactive-DENV (*inact-DV*) serial dilutions (10^{-3} - 10^{-7}) by plaque-forming assay using LLC-MK2 cell. Negative control (C-). Representative image of two experimental replicates.

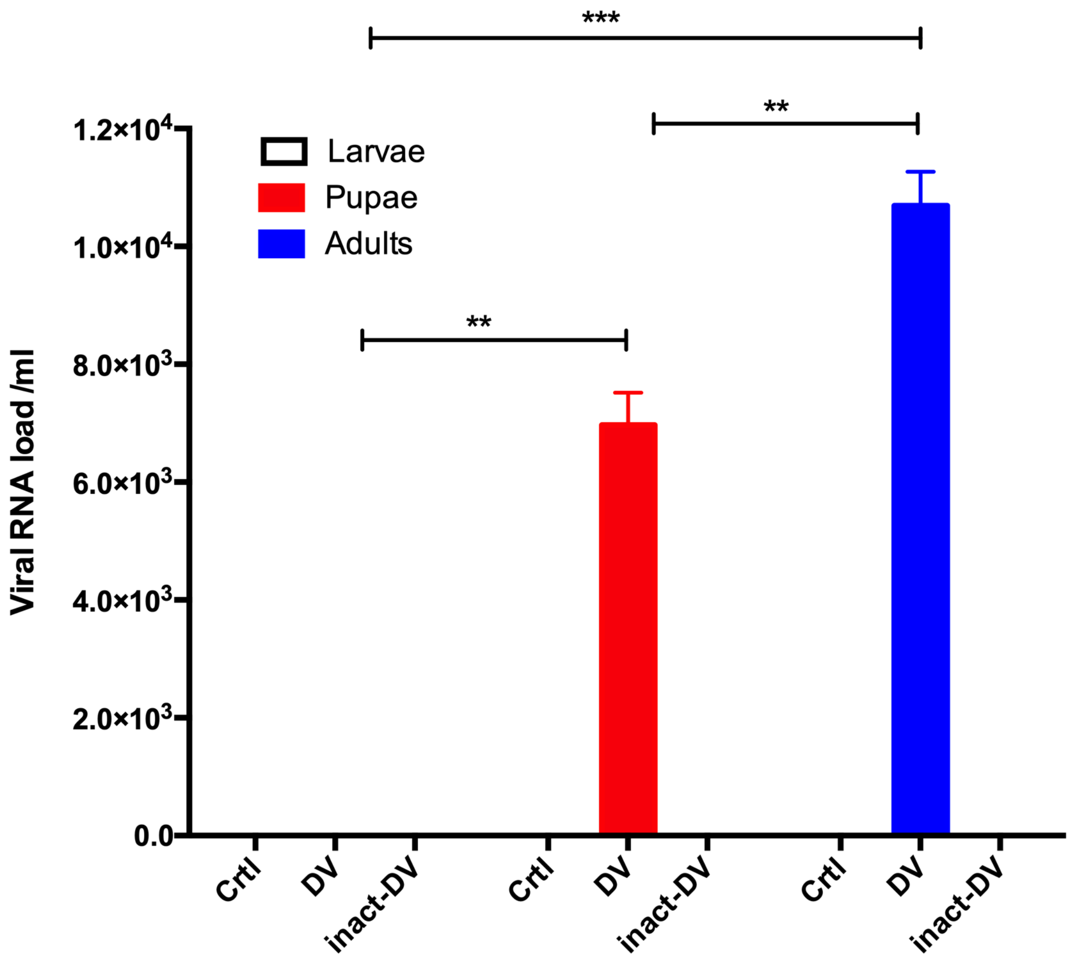

Supplementary Figure 5. Viral RNA load of larvae, pupae and adult mosquitoes (N= 5 individuals per stage) after virus exposure with *DV* (2.1x10^7 PFU/ml) with dilution sterile tap water (proportion 1:100), *inact-DV* (UV) in sterile tap water (1:100) for 24 hours in larvae stage and control group larvae in sterile tap water (*Crtl*). *P*-values represent the statistical significance based on *Mann–Whitney* U-*test*. (***p*<0.01; ****p*<0.001). Values are expressed as the mean ± SE. This experiment was completed at least three times.

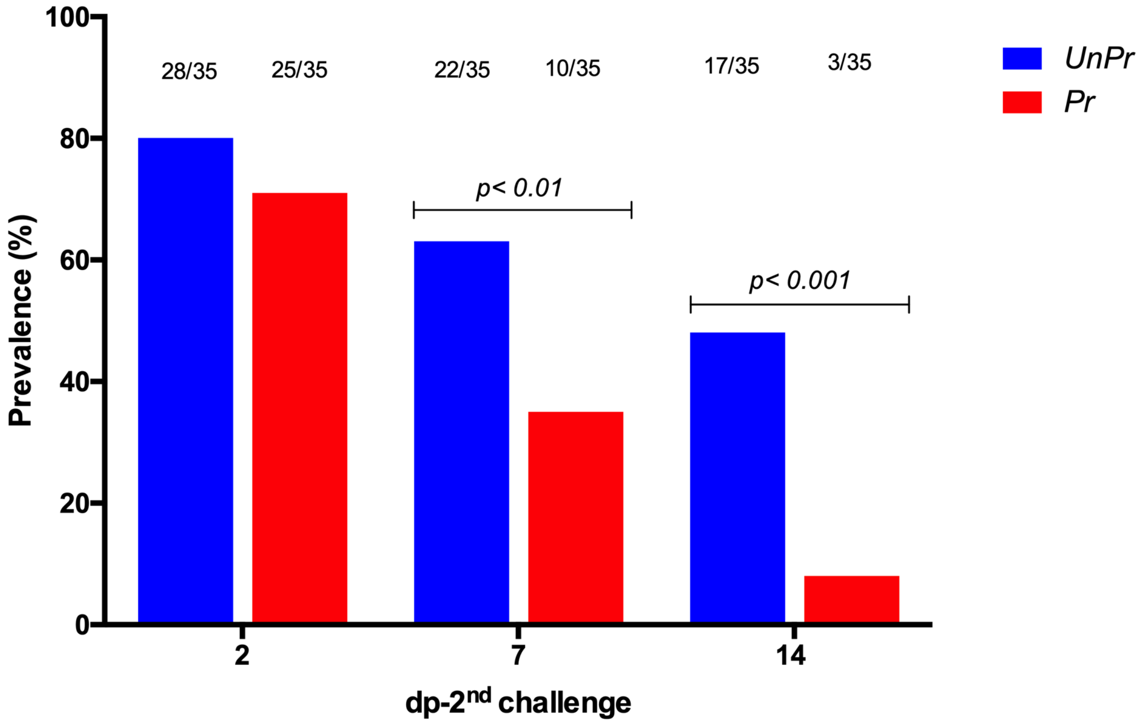

Supplementary Figure 6. Percentage of prevalence of infected mosquitoes for unprimed (*UnPr*) and primed group (*Pr*) at 2, 7 and 14 dp-2nd challenge. *P*-values represent the statistical significance based on *Chi-square statistical test*. The number of infected mosquitoes relative to total mosquitoes is shown at the top of each column. This experiment was repeat at least three times.

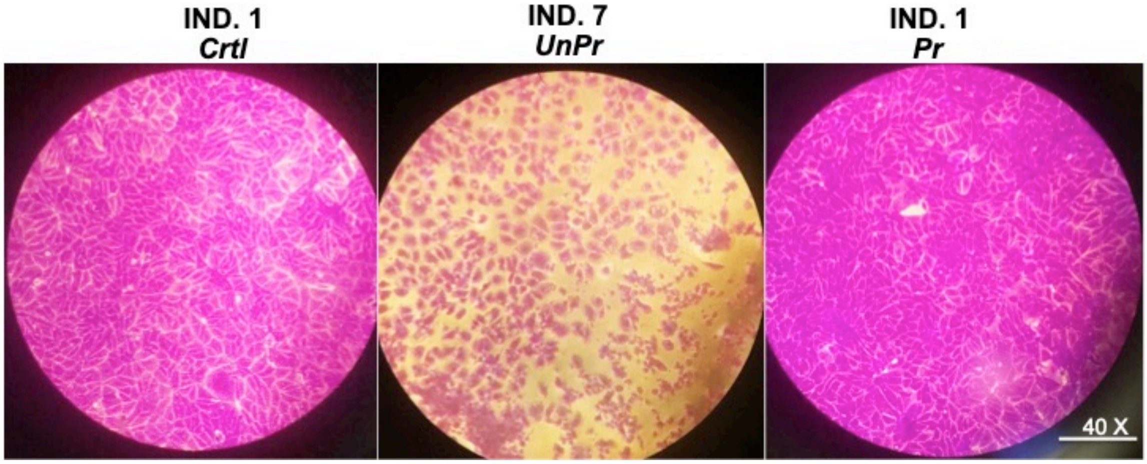

Supplementary Figure 7. Plaque-forming assay using LLC-MK2 cell of individual head mosquitoes for each treatments. Mosquitoes that were fed rabbit blood (*Crtl*); Unprimed mosquitoes (*UnPr*) and primed mosquitoes (*Pr*). Representative images of three experimental replicates of ten individual mosquitoes per group.

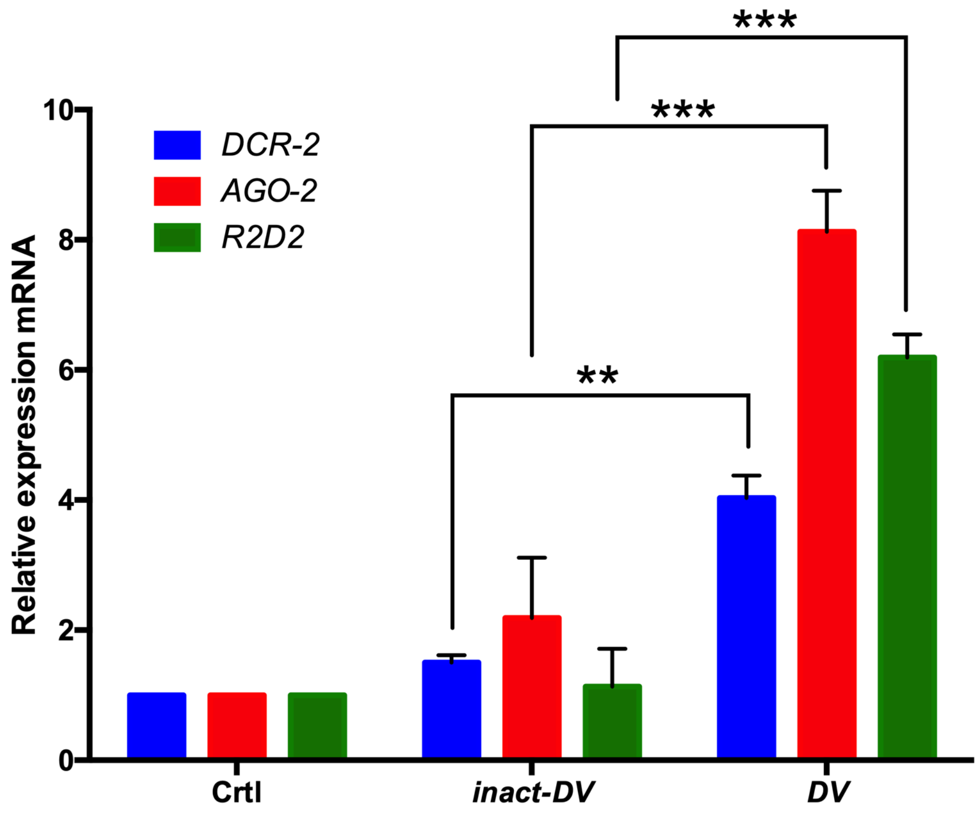

Supplementary Figure 8. Relative expression of *DCR-2*, *AGO-2* and *R2D2* in larvae, after exposed with *DV* ($2.1x10^5$ PFU/ml) and *inact-DV* in larvae stage for 24 hours. Crlt group was exposed only with sterile lap water. *P*-values represent the statistical significance based on *Mann–Whitney* U-*test*. (**p<0.01; ***p<0.001). Values are expressed as the mean ± SE. The data presented represents at least three experimental replicates.

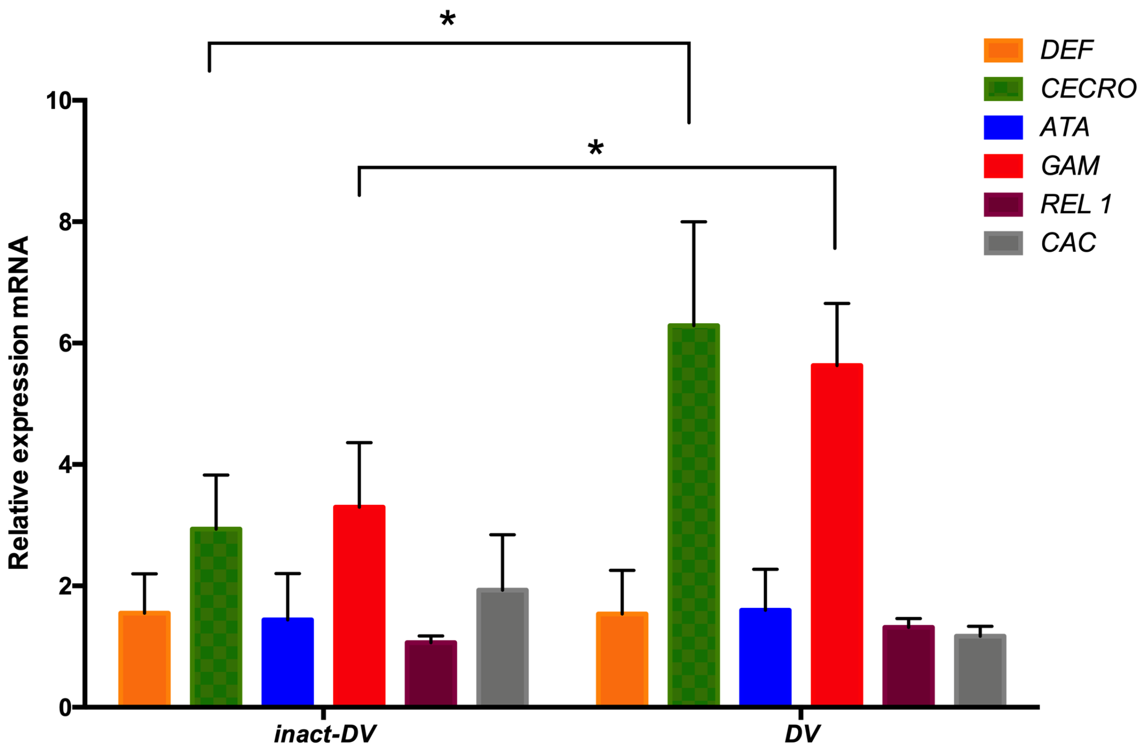

Supplementary Figure 9. Relative expression of *DEF* (Defesin), *CECRO* (Cecropin), *ATA* (Attacin), *GAM* (Gambicin), *REL 1* (Relish 1) and *CAC* (Cactus) in larvae, after exposed with *DV* (2.1×10^5 PFU/ml) and *inact-DV* in larvae stage for 24 hours. *P*-values represent the statistical significance based on *Mann–Whitney* U-*test*. (*$p<0.05$). Values are expressed as the mean ± SE. The data presented represents at least three experimental replicates.

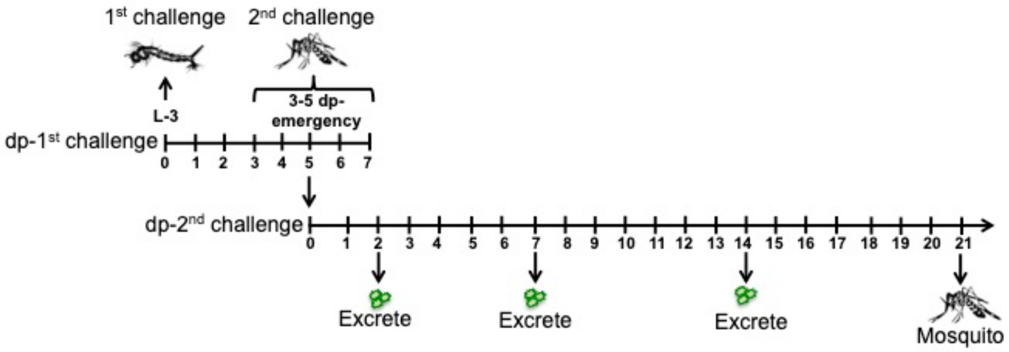

Supplementary Figure 10. Time line of experimental design of days post first challenge (dp-1st challenge) in 3th instar larvae (L-3) and days post second challenge (dp-2nd challenge) in adult mosquitoes at 3 - 5 days post emergency (3 - 5 dp-emergency). Excreta individual mosquitoes were collected at 2, 7 and 14 dp-2nd challenge, while at 21 dp-2nd challenge were collected head and abdomen of individual mosquitoes.

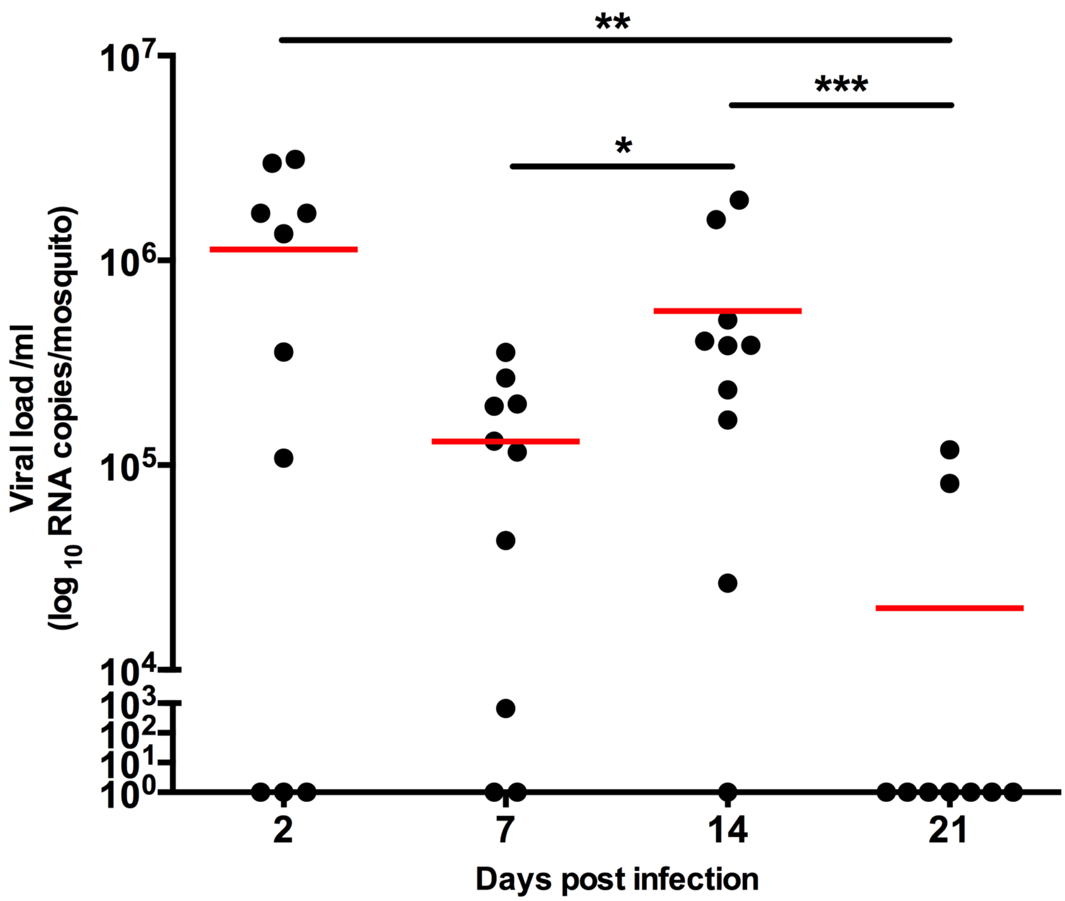

Supplementary Figure 11. Kinetic infection of *DV* in excreta from individual mosquitoes of *Ae. aegypti* (N=10) at 2, 7, 14 and 21 days post infection. *P*-values represent the statistical significance based on *Mann–Whitney* U-*test*. (*p<0.05; **p<0.01; ***p<0.001). Values are expressed as the mean. The data presented represents at least three experimental replicates.
